# REDESIGNING HOSPITAL WORKFLOW TO INCLUDE FAMILY CAREGIVERS OF PEOPLE LIVING WITH DEMENTIA

**DOI:** 10.1093/geroni/igad104.0561

**Published:** 2023-12-21

**Authors:** Catherine Still, Andrea Yahr, Kathryn Istvanek, Andrea Strayer, Nicole Werner, Beth Fields

**Affiliations:** University of Wisconsin-Madison, Madison, Wisconsin, United States; University of Wisconsin-Madison, Madison, Wisconsin, United States; University of Wisconsin-Madison, Madison, Wisconsin, United States; University of Iowa, Iowa City, Iowa, United States; Indiana University School of Public Health-Bloomington, Bloomington, Indiana, United States; University of Wisconsin-Madison, Madison, Wisconsin, United States

## Abstract

Family caregivers of hospitalized people living with dementia (PLWD) report feeling unprepared to fulfill caregiving tasks post hospitalization. Currently, clinicians lack guidance on how to include caregivers in hospital care. This descriptive study used a systems-engineering approach to identify optimal hospital workflows for caregiver inclusion. We conducted 28 hours of direct observations in a large academic hospital with field notes and audio-recorded three hours of interdisciplinary team debriefing meetings. Data analysis was guided by a work system model to map caregiver inclusion workflows using Lucidchart software and identify system barriers and facilitators to caregiver inclusion within each workflow. Results produced workflows of optimal practices for caregiver inclusion during admission, stay, and discharge. Workflow facilitators included healthcare clinicians’ willingness/ability to answer questions, caregiver advocacy skills, and presence of medical record fields focused on patient/family education. Barriers included inconsistent documentation, under-utilization of hospital common spaces, and using a passive approach to caregiver inclusion. Comparison of current practices to desired workflows for caregiver hospital inclusion can provide clinicians guidance on how to identify, assess, and educate caregivers. Future research should determine workflow acceptability and effectiveness for improving caregiver preparedness.

